# Reliability and validity of the Hebrew version of the forgotten joint score for assessing the outcomes of total knee arthroplasty

**DOI:** 10.1186/s42836-021-00084-6

**Published:** 2021-08-03

**Authors:** Amit Pansky, Yaron Bar-Ziv, Eran Tamir, Aharon Finestone, Gabriel Agar, Noam Shohat

**Affiliations:** 1 Sackler School of Medicine, Tel Aviv University, Sharona st’ 14, Ramat Aviv, Rishon le-zion, Israel; 2 Orthopaedic department, Shamir medical center (Assaf Harofe), Rishon le zion, Israel

**Keywords:** Total knee arthroplasty, Validation, Patient-reported outcome, Forgotten joint score, Satisfaction

## Abstract

**Background:**

This prospective study aimed to assess the reliability and validity of the Hebrew version of the forgotten joint score-12 in patients undergoing total knee arthroplasty, because it is going to be used in the Hebrew-speaking populations in Israel.

**Methods:**

The English version of forgotten joint score-12 was translated into Hebrew version by using the standard procedures and in collaboration with its authors. The consecutive patients who had undergone total knee arthroplasty in a single hospital were asked to fill out the Hebrew version of forgotten joint score-12, Oxford knee score, Short Form 12, and visual analog scale. A random subgroup of 60 patients were then asked to fill out a second Hebrew version of forgotten joint score-12 at a minimum of 2-week interval. The reliability was assessed in terms of internal consistency, test-retest reliability and split-half reliability. The validity was measured in terms of the outcomes as mentioned above.

**Results:**

A total of 102 patients participated in the study. The Hebrew version of forgotten joint score-12 showed high reliability. The internal consistency was excellent (Cronbachs’ α = 0.943) and test-retest reliability was high (Intraclass correlation = 0.97). The forgotten joint scores were correlated with the Oxford knee score, Short Form 12, and visual analog scale (*r* = 0.86, *r* = 0.72, and *r*=-0.8, respectively), indicating a high validity.

**Conclusions:**

The Hebrew version of forgotten joint score-12 has excellent reliability, excellent test-retest reliability and good validity. It can be safely used for assessing outcomes of TKA.

## Introduction

Total knee arthroplasty (TKA) represents one of the most common surgeries performed worldwide [1]. However, approximately 20 % of patients report dissatisfaction following primary TKA [2]. The English version of forgotten joint score (FJS)-12 is a self-administered questionnaire for assessing awareness of TKA, but language barrier may pose a challenge when the questionnaire is used in non-English-speaking populations in clinical practice [3–9].

The exact reason for a relatively high dissatisfaction rate is unknown. Many surgeons suggest that it might result from the inability to restore natural joint sensation [9]. Some traditional “surgeon-centered” tools have been developed to assess the outcomes of TKA, including active and passive range of motion, muscle strength, functional tasks, implant survival, etc. [7–9] However, none of the tools assesses patient’s understanding of joint awareness.

In recent years, patient-reported outcomes (PROs) have become increasingly common due to a new insight into the understanding and measuring the surgical outcomes from the patients’ point of view [6]. The forgotten joint score (FJS)-12 was developed by Behrend et al. [9] in 2012. It is a questionnaire based on the notion that a successful surgery enables the patient to be unaware of his artificial joint in daily living. The questionnaire is comprised of 12 self-administered questions regarding joint awareness. It is assumed that the lack of joint awareness implies a successful outcome. It encompasses many factors for a good outcome, including knee pain, mobility, joint stiffness, daily function, and patient expectation. The original English version of FJS-12 shows a high internal consistency (Cronbach *α* = 0.95) and a good correlation with other PROs (r = 0.69–0.79). It also includes a few sociodemographic features that may impact the outcomes. Therefore, a validation study is needed before the FJS-12 is introduced to a new population [9], e.g., a Hebrew-speaking population.

Moreover, because of improved outcomes of TKA and increased patient expectations, many assessments made by the PRO tools result in ceiling effects [10]. In addition, these tools showed the weakness in differentiation between “good” and “excellent” outcomes. The FJS-12 has shown a lower ceiling effect than other PROs, with a strong differentiating power [9]. Its evident effectiveness has made it popular in a great many countries, including Germany, Italy, Spain, Netherlands, France, Poland, Portugal, Sweden, Norway, China, Japan, and the Republic of Korea. Moreover, it has been used as a research tool in more than 180 papers published globally. (http://www.forgotten-joint-score.info/).

The purpose of this prospective study was to examine the reliability and validity of the Hebrew version of the FJS-12 in patients undergoing TKA, since it is going to be used in the Hebrew-speaking populations in Israel.

## Materials and methods

The protocol was approved by the Institutional Review Board that was responsible for human experiments in accordance with the ethical standards. All patients gave informed consent to participate in the study.

The inclusion criteria were patients who had undergone a primary TKA in a single hospital between March 2018 and December 2019, and the patients were sufficiently proficient in Hebrew. The exclusion criteria included another injury or illness of the lower limb, mental disorder, revision TKA, and lack of informed consent.

### English Version of FJS-12

The FJS-12 is a questionnaire consisting of 12 items regarding a patient’s ability to “forget” the artificial joint in everyday life [9]. The 12 questions are about daily living. For each question, there are 6 options, i.e., “never”, “almost never”, “sometimes”, “mostly”, and the last option “irrelevant for me”. The score ranges from 0 to 100, with 100 representing the lowest awareness of the knee implant. If the response to more than 4 items was “non-relevant”, the score should not be used. The English version of FJS-12 is shown in Table 1.

**Table 1:** The English version of the forgotten joint score has 12 questions on the awareness of the artificial joint. The patient ticks one answer from each question

1. … in bed at night?○ never ○ almost never ○ seldom ○ sometimes ○ mostly
2. … when you are sitting on a chair for more than 1 h? ○ never ○ almost never ○ seldom ○ sometimes ○ mostly
3. … when you are walking for more than 15 min? ○ never ○ almost never ○ seldom ○ sometimes ○ mostly
4. … when you are taking a bath/shower? ○ never ○ almost never ○ seldom ○ sometimes ○ mostly
5. … when you are traveling in a car? ○ never ○ almost never ○ seldom ○ sometimes ○ mostly
6. … when you are climbing stairs? ○ never ○ almost never ○ seldom ○ sometimes ○ mostly
7. … when you are walking on uneven ground? ○ never ○ almost never ○ seldom ○ sometimes ○ mostly
8. … when you are standing up from a low-sitting position? ○ never ○ almost never ○ seldom ○ sometimes ○ mostly
9. … when you are standing for long periods of time? ○ never ○ almost never ○ seldom ○ sometimes ○ mostly
10. … when you are doing housework or gardening? ○ never ○ almost never ○ seldom ○ sometimes ○ mostly
11. … when you are taking a walk/hiking? ○ never ○ almost never ○ seldom ○ sometimes ○ mostly
12. … when you are doing your favorite sport? ○ never ○ almost never ○ seldom ○ sometimes ○ mostly

### Translation and Validation

Translation and validation were performed in collaboration with the official developers and according to the accepted guidelines [11] in the following order: (1) preparation of files; (2) two forward translations into the Hebrew language by two independent working translators; (3) reconciliation of these two translations into one optimal version; (4) two back translations of the reconciled version into English; (5) review and discussion of the translated report sent to the developers; (6) proofreading arranged by the developers who sent the results back for approval; (7) pilot testing in 10 patients (10 knees); (8) review of the report; and (9) finalization of the project.

### Patient Evaluation

The research was conducted in a home setting. The patients received a primary call when they were given an explanation regarding the research and asked to give oral consent. Following the consent, the patients were instructed to answer the Hebrew version of FJS-12, Oxford knee score (OKS), 10-cm visual analog scale (VAS), and Short Form (SF)-12 Health Survey. The patients who had undergone staged bilateral knee arthroplasty were asked to answer the questions in terms of the side that received TKA most recently. Sixty patients were randomly selected for assessing the test-retest reliability. After a minimum of 2 weeks following the first call, these patients received a second phone call and were asked to answer the Hebrew version of FJS-12 again. We chose a minimal period of 2 weeks to decrease the patients’ option of remembering the questions. Before the second questionnaire, the patients were asked whether a change in their physical status had occurred.

### Other Assessments

The SF-12 Health Survey Questionnaire is an abridged version of the SF-36 developed in 1996. All 12 items are used to calculate the physical and mental component summary scores by applying a scoring algorithm. The SF-12 can serve as a general tool to evaluate the patients’ general health or well-being following a specific procedure, such as TKA [12, 13]. The SF-12 gives two separate scores (physical and mental). In this study, we assessed only the physical score.

In 1998, the OKS was developed following the Oxford hip score. It is comprised of 12 items, and all are related to the knee joint. Its main application is to assess pain and function in patients with knee osteoarthritis, either before or after surgery. The scores range between 0 and 48 [14]. The VAS was used to rate knee pain in patient’s daily living. The pain was rated on a 1–10 point scale.

We measured the ceiling and floor effects from the percentage of the best or worst possible score [9]. The ceiling and floor effects are commonly accepted when the percentage is less than 15 %. Low ceiling and floor effects indicate a high ability to distinguish between “good” and “excellent” outcomes, which means the questionnaire, as a whole, possesses a high discriminatory power.

### Statistical Analyses

To assess the reliability of the FJS-12, we measured the internal consistency, test-retest reliability, split-half reliability, and the SEM (standard error of measurements) [15]. Internal consistency, measured in Cronbach’s α, tests and confirms a unified construct measured. The scores greater than 0.7 were considered sufficient, scores more than 0.8 were deemed to be good, and scores greater than − 0.9 were considered excellent [16]. The test-retest reliability was assessed in terms of intraclass correlation coefficients (ICC). An ICC greater than 0.7 was considered sufficient [17]. The split-half reliability was rated in terms of the Spearman-Brown coefficient and a value higher than 0.6 was considered adequate [18]. The standard error of the measurement (SEM) was calculated using the following formula: SEM = variance*√(1-ICC) [19]. A small SEM is indicative of high reliability. The smallest detectable change (SDC) was also calculated. SDC is the smallest change in a score that can be interpreted as real change and was calculated by using the formula: SDC = 1.96 * √2 * SEM [19].

Validity is the degree to which the scores of a PRO instrument are consistent with hypotheses based on the assumption that the PRO instrument validly measures the construct to be measured [15]. Validity was measured in terms of the Pearson correlation coefficient with the OKS, SF-12, and VAS. A correlation coefficient was taken as low if it was less than 0.3, moderate if it was in the range of 0.3 to 0.7 and high if it was greater than 0.7.

Additionally, we measured the discriminatory ability of the Hebrew version of FJS-12. We conducted a statistical *t*-test between the high (top 25 %) and low (25 %) score groups for each item on the questionnaire. The statistical significance indicated that the item was able to discriminate between the different groups of patients.

## Results

**Fig. 1:**
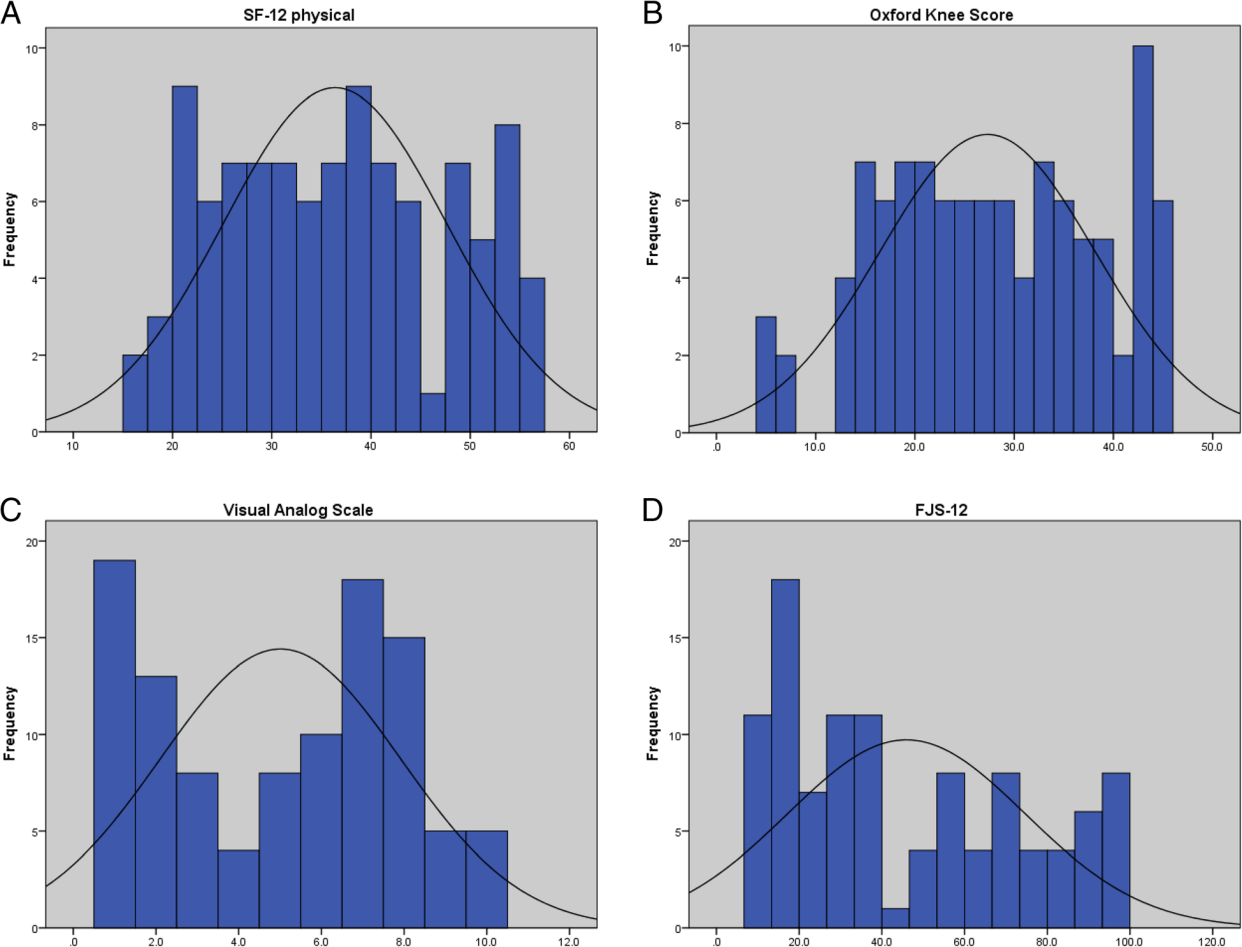
Scatter plot correlating the first and second call on forgotten joint score (FJS)

A total of 110 patients met the inclusion criteria, and all were contacted and agreed to participate in this study. In accordance with the FJS-12 protocol [9], 3 patients were excluded from the analysis because their responses were “irrelevant” with more than 4 items. Five patients refused to participate (95 % acceptance rate). Among them, 2 refused because of privacy concerns and 3 did not provide any answer. One patient completed the first FJS-12 but declined to finish the second FJS-12. The mean patient age was 67.42 ± 7.15 (mean ± standard deviation), 68 (67 %) patients were female, and 34 (33 %) were male. The average follow-up time lasted 12.6 ± 6.47 months. A random group of 60 patients were reached a second time and they responded to the FJS-12 for test-retest reliability (Table 2). The average time interval between calls was 31.26 ± 13.1 days. Only 15 (14 %) patients responded to question number 12 that is related to sports activity, which significantly impacted the evaluation of internal consistency. Therefore, we conducted two internal consistency tests (one covered this question and the other did not). Before exclusion, Cronbach’s α was 0.92, and the inter-item correlation was 0.53. After excluding question 12, the Hebrew version of FJS demonstrated excellent internal consistency with a Cronbach’s α score of 0.943 (95 % confidence interval [CI] 0.92–0.95) and an inter-item correlation coefficient of 0.6. The test-retest reliability was very high, with a measured ICC of 0.97 (95 % CI 0.95–0.98) (Fig. 1). The split-half reliability was increased with a Spearman-Brown coefficient of 0.93 (95 % CI 0.87–0.94); the SEM was 4.97 (low), and accordingly, the SDC was 13.77.

**Table 2:** Demographics of 102 patients (Group 1 did not receive a second call; group 2 received a second call.)

	Overall (*n* = 102)	FJS group 1 (*n* = 42)	FJS group 2 (*n* = 60)
Age (year)	67.42 ± 7.15	65.74 ± 6.72	68.55 ± 7.28
Sex (male : female)	34 (33 %): 68 (67 %)	12 (30 %):28 (70 %)	20 (33 %):40 (68 %)
Follow-up time (months)	12.6 ± 6.4	14 ± 6.74	11.5 ± 6.08
FJS	45.92 ±28.72	43.79 ± 26.32	47.96 ± 29.88

Data were presented as mean ± standard deviation or number (%); FJS, forgotten joint score

**Fig. 2:**
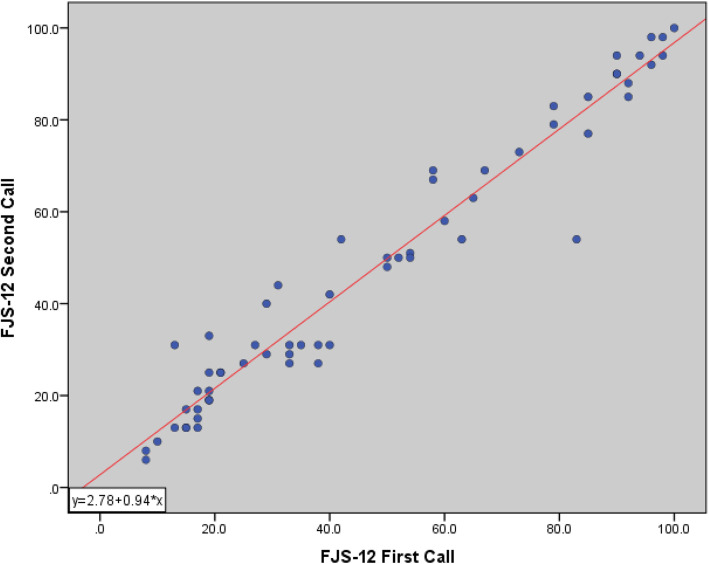
Distributions of forgotten joint score (FJS), Oxford Knee Score (OKS), Short Form Health Survey-12 (SF-12), and visual analog scale (VAS) pain score

Figure 2 shows the score distribution over the scales of the FJS-12, OKS, SF-12 (physical), and VAS pain. The FJS and the OKS showed a positive correlation (*r* = 0.86, *P* < 0.001), which was in the “high” correlation category. The FJS and the SF-12 (physical) also exhibited a positive correlation (*r* = 0.72, *P* < 0.001), which was also in the “high” correlation category. The FJS and the VAS pain score revealed a high negative correlation (*r *= -0.8, *P* < 0.001) (Table 3). Only 2 patients responding to the FJS scored the maximal point of 100, resulting in a negligible ceiling effect of 1.9 %. No patient scored 0, suggesting that there was no floor effect and the FJS-12, as a whole, had high discriminatory power and a good content validity. The *t*-test scores between the high and low scores were greater than 2 (questions 1–11). With all the tests, *P* value < 0.0001.

**Table 3:** Mean scores and correlations with commonly-used patient-reported outcome measures

	FJS-12	OKS	SF-Mental	SF-Physical	VAS	FJS-12: II
Mean ± SD	45.92 ±28.72	27.31 ± 10.85	51.26 ± 11.66	36.36 ± 11.23	5.01 ± 2.90	47.88 ± 29.08
Correlation with FJS-12	1	*r* = 0.86 (high)	*r* = 0.39 (moderate)	*r* = 0.72 (high)	*r*= -0.80 (high)	*r* = 0.97 (very high)
*P* value		< 0.001	< 0.001	< 0.001	< 0.001	< 0.001

*OKS* oxford knee score; *FJS* forgotten joint score; *SF* short form health survey; *VAS* visual analog scale; *SD* standard deviation

## Discussion

TKA procedures have been proven to be highly effective in treating severe osteoarthritis, relieving pain, and restoring joint functionality [4, 7, 8]. The clinical success of the procedure has made it an increasingly common operation. [1, 2]. As a result, nowadays, patients undergoing TKAs are younger and more physically active and the expectation to the procedure is being raised [3]. This trend towards better function and higher success rates, in combination with the shift to patient-centered care, lead to development of multiple PRO tools [6].

These tools have enabled doctors to better evaluate postoperative successes and failures from patients’ perspective and they have showed relatively good discriminatory power. Nonetheless, they lack, as many believe, the critical criteria for judging a successful arthroplasty: a natural joint feeling and joint awareness [9]. What is more, many of these tools have shown considerable ceiling and floor effects, which render it difficult to distinguish between a good and an excellent score [20, 21]. The FJS-12 was developed by Behrend et al. [9] to address these issues. In this study, we were able to reproduce the original paper results, which showed excellent reliability. Moreover, we examined the correlation between the FJS-12 and other commonly-used PROs, which enabled us to show that the Hebrew version of FJS-12 has high validity and is culturally adapted to the Hebrew-speaking populations.

The average patient age in this study was comparable to that of early studies on the subject, and the male-to-female ratio was also similar (the optimal ratio is 2:1) [22, 23]. The sample size was determined by using the recommended guidelines for PRO validation and was applied to each questionnaire item [17]. Notably, question number 12 regarding awareness during physical activity was excluded from the analyses because only 15 (14 %) participants answered this question. This phenomenon was also observed in other Mediterranean countries [23]. The lack of compliance with this question, as compared to all other questions, implies that it is irrelevant with our population. As this work was phone-call based, we understand the reason for non-compliance since most of the subjects do not perform any form of regular physical activity, regardless of their knee status.

The Hebrew version of FJS-12 demonstrated excellent internal consistency with a Cronbach’s α of 0.943, which was virtually identical to the one achieved by Behrend et al. [9] in the original FJS study (having a Cronbach’s α of 0.95). The test-retest reliability was similar to test-retest scores in the early studies conducted in Mediterranean and European countries [23, 24]. The split half-reliability was very high. Finally, the floor effects (zero) and the ceiling effects were significantly lower than the accepted threshold of 15 %, indicating that the entire test has good discriminatory power. Questions 1 to 11 possessed significant discriminatory power.

In this study, we chose to assess the OKS, SF-12, and VAS on the basis of the fact that there is no gold standard for the postoperative evaluation of TKA. In many early studies, different PROs were chosen for comparison. Therefore, it is difficult to compare our outcomes with other studies precisely. Although not comparable, our results still showed a high correlation with different PROs [24–26]. The stronger correlation between the FJS-12 and OKS can be explained by the fact that both questionnaires were explicitly designed to measure knee function during daily activities. The SF-12 measures the general physical function and health, which are influenced not only by TKA outcomes but also by other factors.

In addition, the strong negative correlation of (-0.8) indicates that knee pain is still a significant factor impacting joint awareness. The correlation was negative because a high VAS suggests an undesirable outcome while a high FJS-12 score indicates a desirable outcome.

This study has several limitations. First, we did not assess the responsiveness used for measuring the change in a patient’s condition over time. Second, this study focused only on the postoperative evaluation that the original FJS-12 is designed for, and further assessments are required for understanding the preoperative outcomes. Finally, this study was conducted via phone, which only assessed their ability to understand them, not their ability to read the questions.

## Conclusions

The Hebrew version of FJS-12 has excellent reliability, excellent test-retest reliability, and good validity. It can be safely used for assessing patient outcomes of TKA in the Hebrew-speaking population.

## Data Availability

All necessary data will be provided on demand.

## Abbreviations

TKA: Total knee arthroplasty
ROM: Range of motion
PRO: Patient-reported outcome
FJS: Forgotten joint score
OKS: Oxford knee score
SF 12: Short form 12
VAS: Visual analog score
ICC: Intraclass correlation coefficient
SEM: Standard error of measurement
SDC: Smallest detectable chang
